# The Comprehension of Metaphorical Descriptions Conveying Gender Stereotypes. An Exploratory Study

**DOI:** 10.3389/fpsyg.2019.02615

**Published:** 2019-11-22

**Authors:** Eleonora Borelli, Cristina Cacciari

**Affiliations:** ^1^Department of Biomedical, Metabolic and Neural Sciences, University of Modena and Reggio Emilia, Modena, Italy; ^2^Center for Neuroscience and Neurotechnology, University of Modena and Reggio Emilia, Modena, Italy

**Keywords:** metaphor, gender stereotype, grammatical gender, comprehension, adjective

## Abstract

In this adjective elicitation study, we investigated the comprehension of Italian sentences where a metaphorically intended noun (e.g., *butterfly*, *nightmare*) was used to describe a gender-stereotyped or stereotype-neutral individual (e.g., *flute player*, *engineer, person*). Specifically, we explored whether and to what extent meaning availability and the affective valence of these metaphorical descriptions (e.g., *This flute player is a butterfly*) varied as a function of the stereotypical or stereotype-neutral nature of the sentential subject, the male vs. female direction of the stereotype, and the grammatical gender marked in the subject noun phrase. Our goals were to test whether the meaning of metaphorical descriptions was equally available regardless of the presence and direction of the gender stereotype and of the grammatical gender of the subject, and whether the adjectives expressing the sentential meaning had the same affective valence no matter who was the subject. The results showed that it was easier (i.e., more adjectives came up to mind) to express the sentence meaning when the sentences described male stereotyped individuals than female stereotyped or stereotype-neutral individuals. The adjective valence did not significantly change according to the subject type. Participants produced adjectives with the wrong grammatical gender more often for males in stereotypically female occupations than for females in stereotypically male occupations. These gender errors occurred also when the sentences described females engaged in stereotypically female occupations. Overall, these results extend to metaphorical descriptions previous findings showing that a social group (males) is seen as more *normative* than another (females), and acts as the *unmarked* normative group.

## Introduction

Social stereotypes about race, religion, or gender represent probabilistic generalizations about the attributes of a social group. Specifically, gender-biased stereotypes are associated to actions, attitudes, rules, and other forms of knowledge attributed according to biological gender (for an overview, see Ellemers, 2018). One of the main tools for transmitting gender stereotypes is represented by linguistic expressions. Banaji and Hardin (1996) were among the first to show that many role nouns (e.g., *teacher*, *engineer*) are associated to specific gender-oriented stereotypes. Since then, a wealth of studies (for overviews, see Carreiras et al., 1996; Osterhout et al., 1997; Padovani et al., 2004; Siyanova-Chanturia et al., 2012, 2015) has revealed that the stereotypical gender associated with role nouns is activated immediately (and automatically) as soon as the role noun is read (Pesciarelli et al., 2019), and that this activation is very difficult to suppress (Oakhill et al., 2005).

Large part of the studies investigating gender stereotyping in language tested literal language. But, as we know, this represents only a part of our linguistic repertoire, and maybe not even the most frequent part. According to Ortony (1980), we use figurative language, and notably metaphor, because of its cognitive, interpersonal and communicative impact. The uniqueness of metaphors lies in the fact that they express literally inexpressible concepts; provide a more vivid and image-evoking medium for expressing subjective experiences; and represent a compact form of expression for complex ideas allowing a predication of a many properties in a condensed statement (sometimes a single word).

In everyday language, the use of a metaphorically intended term (e.g., *shark*, *rock*, *iceberg*) to describe a person is rather common. Since the pioneering work of Asch (1958) on double function adjectives (e.g., *cold*), an important, although still poorly understood, question is whether and to what extent a metaphorical or a literal description of a person frame his/her categorization, perception, and stereotyping in similar or different ways (for overviews, see Cacciari, 1998; Maass et al., 2014; Ervas, 2017). To understand a metaphor, we often activate a system of associated commonplaces that do not necessarily represent the “true” of a term (Black, 1979) but rather represents culturally shared conceptual structures. As Glucksberg and Keysar (1990) proposed, in nominal metaphors (i.e., metaphors of the form *A is B*) the metaphorical vehicle (*shark*) refers to a prototypical or ideal exemplar of the category of merciless, aggressive, and predatory individuals, and simultaneously uses that prototype’s name to name this category. These metaphorical descriptions potentially generalize to an entire category (*lawyers*) properties stereotypically thought to be typical of predators (*sharks*). Nominal metaphors can thus allow the generalizations of socially relevant properties across all members of a given category providing a relatively effortless descriptive shortcut. Since metaphors provide a more vivid, condensed and image-evoking medium than plain literal language, a metaphoric framing of social stereotypes can potentially be of greater impact than a literal one since listeners may draw stronger stereotype-consistent inferences from metaphors than from presumably equivalent literal terms (Maass et al., 2014; Ervas, 2017).

Which one of the distinct (although related) meanings of a metaphor is selected by comprehenders depends at the same time on previous context (Gibbs and Colston, 2006; Carston and Wearing, 2011), be it a single word, a sentence, or a long story, and on the interaction between context and the conceptual knowledge associated with the metaphor. For instance, Katz and Pexman (1997) showed that, depending on the characteristics associated to the speaker’s occupation, a same statement was interpreted either as metaphorical or ironical. Statements produced by members of occupations characterized by a large use of irony (high-irony occupations, e.g., comedians) were more likely to be interpreted as ironic and statements produced by members of occupations that often use metaphors (high-metaphor occupations, e.g., priests) as metaphorical. This suggests that interpersonal context and stereotypical beliefs about occupations jointly modulate the interpretation assigned to metaphorical statements.

To the best of our knowledge, few studies experimentally tested the impact of gender stereotypes and metaphors (both linguistic shortcuts) on sentence comprehension (for overviews, see Maass et al., 2014; Ervas, 2017). For instance, in a property elicitation study, Hegstrom and McCarl-Nielsen (2002) asked participants to metaphorically describe familiar persons. The content analysis of these descriptions showed that physical appearance played a major role with women mostly described as cheerful, dependent, and attractive and men as strong or sturdy and big or tall.

## The Present Study

The main aim of this exploratory study was investigating the comprehension of Italian sentences where a metaphorically intended noun (e.g., *butterfly*, *nightmare*) was used to describe a gender-stereotyped (e.g., *flute player*, *engineer*) or a stereotype-neutral individual (e.g., *person*). Specifically, we explored whether the meaning availability and affective valence assigned to metaphorical descriptions were influenced by the stereotypical or stereotype-neutral nature of the sentential subject (e.g., *flute player*, *engineer, person*), and by the gender direction of the stereotype. Since Italian is a gender-marked language, we also tested the effect of the grammatical gender assigned to the sentential subject. In Italian, as in most Romance languages, the masculine and feminine gender of nouns is always specified (neuter is absent), with a conceptual criterion for assigning gender to human beings (mostly based on biological gender) and an arbitrary criterion for assigning gender to objects, abstract entities, and some animals. In addition, some nominal endings (-*a* and –*o*) typically map onto a specific gender class (feminine and masculine, respectively). The nominal ending can be a morphological marker for gender in nouns with a biological gender (e.g., *ragazz-a* “girl”, *ragazz-o* “boy”). Only a small part of Italian nouns, namely bigender nouns, does not follow this criterion since their word ending is not marked for grammatical gender that rather has to be inferred from context or from constituents such as, for instance, a determiner, or a past participle (Cacciari et al., 1997).

The main difference between grammatical and stereotypical gender is that while grammatical information determines the gender of a noun categorically (Masc. vs. Fem. in Italian; Padovani et al., 2004), stereotypical gender information represents a probabilistic bias that in principle can or cannot drive the assignment of a female/male feature to a noun (Canal et al., 2015).

We manipulated the grammatical gender of the determiner (*Questo*_*M*_/*Questa*_*F*_; *This*; *Quello*_*M*_/*Quella*_*F*_; *That*) preceding the bigender role nouns to obtain sentences where the bigender subject was unambiguously marked for gender by the determiner as either referring to a male or to a female individual. This led to descriptions where the grammatical and stereotypical gender of the person matched and descriptions where they mismatched (see below for examples). As a control condition, we used the epicene *persona* as sentential subject. In Italian, epicenes are grammatically marked for gender but can be used to refer to individuals of both biological genders (Cacciari et al., 1997, 2011).

We used conventionalized metaphorical nouns (e.g., *shark*, *rock*) since they represent easy to access, socially shared familiar knowledge structures. Would the masculine or feminine direction of the stereotype affect the meaning availability and the affective valence of the adjectives expressing the sentential meaning? Social psychologists (Eagly and Kite, 1987; Bem, 1993; Hegarty and Pratto, 2001) argued that attitudes, beliefs, and stereotypes are more influenced by male exemplars than female ones. In addition, stereotypically male occupations generally have higher social status and power than female ones (Eagly, 1987; Ridgeway, 2001). Hence, it may be easier to access the properties associated to male than female stereotyped role nouns leading to more adjectives for expressing the meaning of, for instance, *This parachutist is a rock* than *This teacher is a rock*.

This unbalanced social weight of male and female stereotypes may also affect the valence of the adjectives expressing the sentence meaning, and even more so when biological and stereotypical gender mismatch. Indeed, all other things being equal, often socially *atypical* female and male individuals (that is individuals engaged in occupations stereotypically typical of the other gender as, for instance, female engineers or male teachers) are judged more negatively than socially *typical* ones (male engineers or female teachers; *Backlash effect*; Rudman and Glick, 2001; Phelan et al., 2008). In addition, previous psycholinguistic and ERP studies (for overviews, see Siyanova-Chanturia et al., 2012, 2015; Mado Proverbio et al., 2018) showed asymmetrical effects in the comprehension of male and female stereotyped role nouns. For instance, in Cacciari and Padovani (2007) a male stereotyped role noun (*engineer*) facilitated the gender classification of a masculine pronoun (*He*) and did not interfere with the decision about a feminine pronoun (*She*). In contrast, a female stereotyped role noun (*teacher*) facilitated the gender decision about *She* but disrupted the gender decision about *He*. In Siyanova-Chanturia et al. (2012) gender stereotypes were less restrictive for females than males since participants were more accepting of female drivers (a male stereotyped occupation) than of male teachers (a female stereotyped occupation). Does this reflect the fact that there are, for instance, more female teachers than male ones? Interestingly, numerical prevalence does not seem to be the main factor (Miller et al., 1991) since the categories for which men are thought to be prototypical exemplars do not necessarily contain more men than women.

Finally, we also tested whether the participants’ reading habits influence meaning availability based on previous evidence that the comprehension of figurative language is affected by the width of semantic knowledge (for an overview, see Cacciari et al., 2018).

## Materials and Methods

### Participants

One hundred sixty four undergraduates volunteered to participate in this online study (134 females, mean age = 24.3, SD = 6.6). Forty seven different participants (34 females, mean age = 32.3, SD = 4.6) volunteered to participate in the norming phase and 108 (77 females, mean age = 28.3; SD = 8.17) volunteered to participate in the valence rating of adjectives. They were all Italian native speakers. The study was performed in accordance with the ethical standards of the 2013 version of the Declaration of Helsinki, with the recommendations of the Italian Association of Psychology (AIP) Ethical Guidelines (Codice Etico: www.aipass.org/node/11560), and with the standard ethical procedures adopted by the University of Modena and Reggio Emilia.

### Materials

#### Nouns in the Subject Position

Sixty Italian role nouns either associated to stereotypically male occupations (*N* = 30) or stereotypically female occupations (*N* = 30) were selected from Misersky et al. (2014) and Fabre et al. (2015). All role nouns were bigender, that is the word form did not convey any grammatical or morphological cue to gender (e.g., *autista, ingegnere* for male-oriented stereotypes; *babysitter, insegnante* for female-oriented stereotypes). Stereotypically male and female role nouns were balanced for strength of stereotypical association, valence, wealth, length (number of characters) and Google written frequency, but not for social status that was slightly higher for stereotypically male than female occupations (see Table 1 for mean values and statistics). The mean ratings of stereotypical association, valence, wealth, and length were taken from Fabre et al. (2015) and were instead obtained *de novo* for the 8 role nouns extracted from Misersky et al. (2014) by asking 19 participants (13 females, mean age = 30.3, SD = 4.1) to rate each noun using the same scales of Fabre et al. (2015). Google written frequency was calculated at the time of the norming. The control condition was represented by the epicene word *persona*. Since, as we said, in Italian the grammatical gender of the determiner unambiguously establishes the gender of the following constituent, the role nouns were preceded by grammatical gender marked singular determiners (*Questa/o*, *Quello/a*).

**TABLE 1:** Mean ratings of the psycholinguistic characteristics of stereotypical role nouns (standard deviations in brackets). Length: Number of characters.

	**Stereotype strength**	**Valence**	**Wealth**	**Social Status**	**Length**	**Google LogFrequency**
Male stereotypes	2.56 (0.51)	4.56 (0.78)	4.11 (0.92)	4.32 (1.04)	8.77 (1.99)	6.92 (1.0)
Female stereotypes	3.1 (0.95)	4.45 (0.90)	3.87 (0.96)	3.61 (0.99)	9.77 (2.22)	6.32 (0.84)

*Rating scales: From 1 (Only for males; Very negative; Very rich occupation; Very low social status) to 7 (Only for females; Very positive; Very poor occupation; Very high social status).*

#### Metaphorical Vehicles

Thirty nouns to be used as metaphorical vehicles were selected from Cacciari et al. (2018). Fourteen of them had a positive valence and 16 a negative one. Their mean ratings of polysemy, length, age of acquisition (AoA), imageability and concreteness are shown in Table 2.

**TABLE 2:** Mean ratings of the psycholinguistic characteristics of the nouns used as metaphorical vehicles (standard deviations in brackets).

	**Polysemy**	**Length**	**AoA**	**Imageability**	**Concreteness**
Nouns	5.09 (3.12)	7.1 (1.4)	3.57 (1.2)	5.31 (0.98)	2.05 (1.05)

*Polysemy: Mean number of meanings listed in Italian Dictionaries. Rating scales: From 1 (Year 0–2; Easy to imagine; Concrete) to 7 (Year 13 and beyond; Difficult to imagine; Abstract).*

#### Metaphorical Word Pairs

Ratings of familiarity, aptness, comprehensibility, and imageability of neutral, stereotypically male and female word pairs were obtained by asking 28 participants (21 females, Mean age = 34.3, SD = 3.03) to rate each pair (e.g., *flute player-butterfly*) (see Table 3). We also collected Google written frequency for each word pair. Since we did not have an *a priori* hypothesis about the distributional properties of the word pairs, we did not include any specific word distance in the Google frequency search of the word pair co-occurrence. Surprisingly, the sentences introduced by *persona* (neutral condition) were more frequent, and were rated as more familiar, apt, comprehensible, and imageable than those introduced by stereotypically male and female occupation nouns that did not significantly differ (see the Appendix for the word pairs). This may reflect the generic nature of the subject *persona* that led participants to interpret each sentence without considering the constraints induced by a specific role noun, as if they had mentally listed all the possible meanings that a metaphor can have.

**TABLE 3:** Mean ratings of the psycholinguistic characteristics of the metaphorical word pairs (standard deviations in brackets).

	**Familiarity**	**Aptness**	**Comprehensibility**	**Imageability**	**Google LogFrequency**
Male stereotypes	2.97 (1.47)	2.48 (0.87)	3.64 (1.52)	3.57 (1.45)	4.96 (1.0)
Female stereotypes	2.89 (1.33)	2.32 (0.88)	3.66 (1.43)	3.62 (1.38)	4.52 (0.94)
Control condition	5.24 (1.12)	3.86 (0.59)	5.45 (1.0)	5.42 (0.86)	5.84 (0.6)

*Rating scales: From 1 (Not at all familiar; The meaning of vehicle does not convey an important aspect of the subject; Not at all comprehensible; Easy to imagine) to 7 (Extremely familiar; The meaning of vehicle conveys an extremely important aspect of the tenor; Extremely comprehensible; Difficult to imagine).*

Subject-metaphorical vehicle pairs were embedded in well-formed nominal sentences starting with a determiner grammatically marked for gender (*Questa/o*; *Quello/a*). Since in Italian the grammatical gender of the adjective must agree with the biological gender of the individual it refers to, the presence of a grammatically feminine determiner implies that the sentence concerns a female individual and a grammatically masculine determiner a male individual. We had the following five conditions (Table 4):

**TABLE 4:** Experimental conditions and examples.

**Gender direction of the stereotype**	**Grammatical gender marked in the determiner**	**Experimental conditions**	**Example**
Male stereotypes	Masculine gender	Male-congruent condition	“This_*M*_ land-surveyor is an iceberg”
	Feminine gender	Male-incongruent condition	“This_*F*_ land-surveyor is an iceberg”
Female stereotypes	Feminine gender	Female-congruent condition	“This_*F*_ assistant is an iceberg”
	Masculine gender	Female-incongruent condition	“This_*M*_ assistant is an iceberg”
Control condition	Neutral condition	Control condition	“This person is an iceberg”

(1) Control condition not semantically or stereotypically marked for gender (e.g., *Questa persona è un iceberg*, *This person is an iceberg*);
(2) Male-congruent condition: the grammatical gender of the subject, as introduced by the grammatical gender marking of the determiner, matched the masculine direction of the stereotype (GSM congruent; e.g., *Questo geometra è un iceberg*, *This_*M*_ land-surveyor is an iceberg*);
(3) Male-incongruent condition: the grammatical gender of the subject mismatched the masculine direction of the stereotype (GSM incongruent; e.g., *Questa geometra è un iceberg*, *This_*F*_ land-surveyor is an iceberg*);
(4) Female-congruent condition: the grammatical gender of the subject matched the feminine direction of the stereotype (GSF congruent; e.g., *Questa assistente è un iceberg*, *This_*F*_ assistant is an iceberg*);
(5) Female-incongruent condition: the grammatical gender of the subject mismatched the feminine direction of the stereotype (GSF incongruent; e.g., *Questo assistente è un iceberg*, *This_*M*_ assistant is an iceberg*).

The resulting 150 sentences were split into two lists each containing the same number of sentences per condition (75 sentences). The participant task was to write down up to three adjectives expressing the sentential meaning. Each participant responded only to one list in which s/he saw the same metaphorical vehicle in the five conditions but never one after the other. The order of the sentences was quasi-randomized.

To assess the participants’ reading habits, we designed a questionnaire, modeled after the questionnaires typically used in the literature, in which we asked participants to specify how much they liked reading; how many books (excluding textbooks) in a year; the preferred genres; what they liked reading aside books; and whether they preferred reading on paper or using an electronic support. Designing an *ad hoc* questionnaire was motivated by the lack of a standardized measure of reading habits in Italian.

### Procedure

Participants received an e-mail asking whether they were willing to participate in a web survey. The e-mail also contained instructions on how to access a randomly assigned, self-paced anonymous questionnaire via Survey Monkey. Each questionnaire started with an introduction explaining that the study aimed at investigating the ways in which we describe an individual. This was followed by instructions specifying how to carry out the task and some examples of sentences (different from the experimental ones). The instructions were as follows (we present a word-by-word English translation): “You will find a list of sentences that may be used to describe people (for example: “That person is a chameleon”). For each sentence, we ask you to list three adjectives that in your opinion can express the sentence meaning (for example: for “That person is a chameleon” adjectives such as “changing”, “turncoat”, “resilient”). If you cannot come up with three adjectives, please provide at least one. At the end of the questionnaire, we ask you to respond to some questions about your reading habits.”. The questionnaire also asked for demographic information (i.e., gender, age, mother tongue, profession, and education).

### Data Analysis

We analyzed the percentage of grammatically correct adjectives and of grammatically incorrect (i.e., disagreeing) adjectives provided for each metaphorical sentence in each condition. Grammatical disagreeing adjectives are adjectives that do not match with the gender marked by the determiner in the metaphor.

The statistical analyses were conducted on the by-item percentages of response in each experimental condition using Bonferroni corrected *t*-tests. Partial correlation analyses were also carried out between the characteristics of the role nouns (frequency, social status, wealth, and stereotype strength) and the percentages of correct and incorrect adjectives in the five conditions. The answers to the reading habits questionnaire were recoded assigning numerical values to each response type for each subject (i.e., I like reading: very much = 4, enough = 3, not much = 2, not at all = 1; Number of book read in a year: 0–2 = 1, 3–4 = 2, 5–6 = 3, 7–8 = 4, 9–10 = 5, beyond 10 = 6, Education: secondary school = 1, high school = 2, graduation = 3, beyond = 4). By-subject bivariate correlations were calculated between each type of reading habit recoded answer and the total number of correct and incorrect adjectives. *t*-tests were also used to assess possible differences in the valence mean ratings of the first adjective provided for each of the 150 sentences.

## Results

One hundred sixty four questionnaires were completely and accurately responded to. Overall participants listed 26454 adjectives (out of the expected 36900), 87.4% of which were grammatically correct, 0.8% contained grammatical gender agreement errors, and 0.5% were verbs or short sentences rather than adjectives and therefore they were not further analyzed (Table 5).^1^

**TABLE 5:** Mean percentage of grammatically agreeing and disagreeing adjectives and of other types of response by condition and congruency between grammatical (G) and stereotypical gender (S) (standard deviations in brackets).

	**Agreeing adjectives**		**Disagreeing adjectives**		**Other responses**	
	**GS congruent**	**GS incongruent**	**GS congruent**	**GS incongruent**	**GS congruent**	**GS incongruent**
Male stereotypes	91.5 (4.5)	75.1 (9.5)	1.9 (2.0)	18.6 (10.3)	6.5 (3.9)	6.2 (4.5)
Female stereotypes	82.3 (7.7)	84.7 (10.2)	11.7 (6.9)	8.0 (10.5)	6.0 (4.2)	7.3 (4.3)
Control condition	86.4 (7.4)		7.7 (6.5)		6.0 (2.8)	

*t*-tests on the by-item percentage of the grammatically agreeing adjectives in the five experimental conditions were carried out (Bonferroni corrected for multiple comparisons, 0.05/8 α = 0.006). We only report significant effects that were:

(1) A significantly higher percentage of adjectives when a stereotypically male role noun was preceded by a stereotype-congruent determiner than by an incongruent one (*This_*M*_ land-surveyor vs. This_*F*_ land-surveyor is an iceber*g) [*M* = 91%, SD 4.5% vs. *M* = 75%, SD 9%, respectively; *t*(29) = 7.711, *p* = 0.0001];
(2) A significantly higher percentage of adjectives when a stereotypically male role noun was preceded by a stereotype-congruent determiner than when a stereotypically female role noun was preceded by a stereotype-congruent determiner (*This_*M*_ land-surveyor* vs. *This_*F*_ assistant*…) [*M* = 91%, SD 4.5% vs. M = 82%, SD 8%, respectively; *t*(29) = 6.131, *p* = 0.0001];
(3) A significantly lower percentage of adjectives when a stereotypically male role noun was preceded by a stereotype-incongruent determiner than when a stereotypically female role noun was preceded by a stereotype-incongruent determiner (*This_*F*_ land-surveyor* vs. *This_*M*_ assistant*…) [*M* = 75%, SD 9.5% vs. *M* = 86%, SD 7%; *t*(29) = 4.118, *p* = 0.0001];
(4) A significantly higher percentage of adjectives when the subject was a stereotypically male role noun preceded by a stereotype-congruent determiner than when it was stereotype-neutral [*M* = 91%, SD 4.5% vs. *M* = 86%, SD 7%; *t* (29) = 3.558, *p* = 0.001];
(5) A significantly lower percentage of adjectives when the subject was a stereotypically female role noun preceded by a stereotype-congruent determiner than when it was stereotype-neutral [*M* = 82%, SD 8% vs. *M* = 86%, SD 7%; *t* (29) = 3.004, *p* = 0.005].

As to errors (i.e., grammatically disagreeing adjectives), the highest percentage of first position disagreeing adjectives occurred when stereotypically male role nouns were preceded by stereotype-incongruent feminine determiners (*This_*F*_ land-surveyor*…) (*M* = 19.0%, SD 10.3%). Surprisingly, grammatically disagreeing adjectives also occurred when stereotypically female role nouns were preceded by grammatically feminine determiners (*This_*F*_ assistant*…) (*M* = 11.7%, SD 6.8%), namely when grammatical and stereotypical gender matched.

*t*-tests on the by-item percentage of grammatical agreement errors (i.e., adjectives marked with the wrong grammatical gender) in the five experimental conditions were carried out (Bonferroni corrected for multiple comparisons, 0.05/8 α = 0.006). We only report significant effects that were:

(1) A significantly higher percentage of errors when a stereotypically male role noun was preceded by a grammatically incongruent than by a congruent determiner [*M* = 18.6%, SD 10.3% vs. *M* = 1.9%, SD 2%; *t*(29) = 8.256, *p* = 0.0001];
(2) A significantly higher percentage of errors when the subject was stereotype-neutral than when it was a stereotypically male role noun preceded by a grammatically congruent determiner [*M* = 7.7%, SD 6.5% vs. *M* = 1.9%, SD 2%; *t*(29) = 4.64, *p* = 0.0001];
(3) A significantly lower percentage of errors when the subject was stereotype-neutral than when it was a stereotypically male role noun preceded by a grammatically incongruent determiner [*M* = 7.7% vs. *M* = 18.6%, SD 10.3%; *t*(29) = 4.898, *p* = 0.0001];
(4) A significantly higher percentage of errors when a stereotypically female noun was preceded by a grammatically congruent determiner than when a stereotypically male noun was preceded by a grammatically congruent determiner [*M* = 11.7%, SD 6.9% vs. *M* = 1.9%, SD 2%; *t*(29) = 7.478, *p* = 0.0001];
(5) A significantly higher percentage of agreement errors when a stereotypically male noun was preceded by a grammatically incongruent determiner than when a stereotypically female noun was preceded by a grammatically incongruent determiner [*M* = 18.6%, SD 10.3% vs. *M* = 8.0%, SD 10.5%; *t*(29) = 3.922, *p* = 0.0001].

Interestingly, partial correlation analyses considering the characteristics of the role nouns (frequency, social status, wealth and stereotype strength) and the mean percentage of correct and incorrect adjectives in the five conditions revealed that grammatical gender agreement errors were influenced by the social status associated to the role nouns but only for male-oriented stereotypes in the congruent condition: the higher the social status, the fewer the gender disagreeing adjectives [*r* (df 24) = −0.529, *p* = 0.007)].

To assess the affective valence of adjectives, we selected the first adjective most frequently produced for each of the 150 sentences. This led to 159 adjectives that were randomly divided into three lists presented via Survey Monkey to 108 participants (77 females, mean age = 28.3, SD = 8.2). They were asked to rate the valence of each adjective with a rating scale going from 1 (*Extremely negative*) to 7 (*Extremely positive*). At variance with our hypothesis, there were no significant effects of condition, with mean valence ratings ranging from 3.9 to 4.2 (see Tables 6, 7). The adjectives produced for stereotypically male descriptions were always slightly more positive than the adjectives produced for stereotypically female and stereotype-neutral descriptions, but none of these differences were statistically significant. In sum, grammatical gender and stereotype direction did not significantly modulate the perceived affective valence of the adjectives. However, this lack of significant effects of valence, that counters our hypothesis, may reflect the fact that the adjective valence was assessed by a different pool of participants and out of context, since participants rated the adjectives without the sentences they refer to.

**TABLE 6:** Mean valence of the first adjective by condition and congruency between grammatical (G) and stereotypical gender (S) (standard deviations in brackets).

	**Valence rating**	
	**GS congruent**	**GS incongruent**
Male stereotypes	4.22 (1.5)	4.23 (1.6)
Female stereotypes	3.9 (1.7)	4.05 (1.7)
Control condition	4.07 (1.6)	

**TABLE 7:** *t*-tests on valence ratings by condition and congruency between grammatical (G) and stereotypical gender (S).

**Experimental conditions**	***t*-test**
Male stereotypes GS congruent vs. Incongruent	*t*(df 29) = 0.303, *p* = 0.754
Female stereotypes GS congruent vs. Incongruent	*t*(df 29) = 1.034, *p* = 0.31
Male stereotypes GS congruent vs. Female stereotypes GS congruent	*t*(df 58) = 0.789, *p* = 0.434
Male stereotypes GS incongruent vs. Female stereotypes GS incongruent	*t*(df 58) = 0.622, *p* = 0.537
Male stereotypes GS congruent vs. Control condition	*t*(df 58) = 0.363, *p* = 0.718
Female stereotypes GS congruent vs. Control condition	*t*(df 58) = 0.587, *p* = 0.56

Finally, we analyzed whether the participants’ reading habits influenced the results of the adjective elicitation task, but none of the correlations between the by-participant mean values in each item of the reading habit questionnaire and the mean percentage of correct and grammatically incorrect adjectives was statistically significant (*Total number of correct adjectives* with *Education*: *r* = 0.008, df 164, *p* = 0.918; with *How much I like reading*: *r* = 0.045, df 163, *p* = 0.564; with *Number of books read*: *r* = 0.004, df 164, *p* = 0.962; *Total number of agreement errors* with *Education*: *r* = −0.004, df 164, *p* = 0.956; with *How much I like reading*: *r* = −0.106, df 163, *p* = 0.179; and with *Number of books read*: *r* = −0.043, df 164, *p* = 0.587). The only significant correlation concerned reading habits *per se* in that the more participants liked to read and the more books they reported to have read [*r* = 0.649, (df 164), *p* = 0.0001]. 60.4% of the participants indicated that they liked reading a lot and 30.4% enough. Excluding textbooks, 16.5% declared to read between 0 and 2 books per year, 47.6% between 3 and 8 books, and 25.6% beyond 10. Their preferred literary genres were fantasy (48.8% of the responses), followed by adventures (44.5%), sentimental (40.8%), mystery (39.6%), thriller (39.2%), science fiction (28.4%) and biography (26.8%). Participants declared to read also online news (73.2%), followed by journals (45.7%), cartoons (37.2%), daily newspapers (31.7%) and online narrative (29.9%). Books were preferentially read in a paper format than via an electronic support (93.9 vs. 6%, respectively).

## Discussion

Metaphors do not concern only language and communication. Rather, since metaphors instantiate the cultural models of the world we live in Lakoff and Johnson (1980), Quinn (1991), Gibbs and Colston (2006), Landau et al. (2014), structure the ways in which we see ourselves, the inner and outer worlds (for overviews, see Glucksberg, 2001; Gibbs, 2008), they can provide an interesting test case for investigating the interplay between social stereotypes and their linguistic expression.

The basic questions motivating this exploratory study concerned the comprehension of sentences where metaphorically intended terms were used to describe individuals characterized by a gender stereotyped role noun (e.g., *This flute player is a butterfly*) or a stereotype-neutral noun (e.g., *This person is a butterfly*). Specifically, we wondered whether the meaning of metaphorical descriptions would be equally available when the sentential subject was expressed by a male- or female-oriented gender stereotype, and whether a same metaphorical descriptor (*butterfly*) would have a different affective valence when used to describe a pilot or a flute player. Since Italian determiners are grammatically marked for gender, we also tested effects of grammatical gender that are well attested in the gender stereotype literature (for an overview, see Molinaro et al., 2016).

More adjectives came to mind for sentences describing male stereotyped individuals than female stereotyped and stereotype-neutral individuals. More adjectives also were produced for sentences with stereotypically male role nouns than female ones when both were preceded by grammatically congruent determiners. A lower meaning availability (i.e., fewer adjectives) was instead observed when stereotypically male role nouns were preceded by feminine determiners and when the subjects were described with stereotypically female role nouns compared to the neutral condition. In sum, in line with our prediction, it was easier (i.e., more adjectives came to mind) to express the meaning of sentences describing male stereotyped individuals than female stereotyped or stereotype-neutral individuals (Eagly, 1987; Ridgeway, 2001). Overall, this provides further evidence that male-oriented stereotypes indeed are easier to access than female-oriented stereotypes and tend to be more normative (for an overview, see Garnham et al., 2017). But while previous results were obtained predominantly using literal person descriptions, the present study offers new evidence extending these effects to metaphorical descriptions.

Why fewer adjectives were available to describe the meaning of sentences describing females occupied in stereotypically male occupations than males in stereotypically female occupations (*This_*F*_ judge*… vs. *This_*M*_ assistant*…)? One possibility is that the *Backlash effect* (Rudman and Glick, 2001; Phelan et al., 2008) may be gender-asymmetrical with the properties associated to female *atypical* descriptions (e.g., *judge*) less easy to retrieve than those associated to male *atypical* descriptions (e.g., *assistant*). Another possibility is that participants considered males as the unmarked group that includes females as well. This would be consistent with the fact that traditionally, in Italian, females are described using the grammatically masculine form of the occupation noun (e.g., *avvocato*_*M*_, *lawyer*) (although this habit is changing). In addition, it may reflect the actual distribution of these occupations in Italy or at least the way in which it is perceived. Future studies are needed to assess the roles of these not necessarily alternative possibilities.

Interestingly, agreement errors (i.e., adjectives marked with the wrong grammatical gender) revealed effects of gender stereotypes as well. The highest percentage of agreement errors occurred when a stereotypically male occupational noun was associated with a female individual (*This_*F*_ judge is a* …). Adjectives were marked with the wrong grammatical gender more often when referring to males in stereotypically female occupations (*This_*M*_ assistant is a*…) than females in stereotypically male occupations (*This_*F*_ judge is a*…). Agreement errors occurred even when stereotypically female nouns were congruently associated with females, again suggesting that indeed males, as a social group, represent the unmarked normative group. Overall, these agreement errors suggest, together with meaning availability differences, that one group (male individuals) tend to be considered as more inclusive than the other group to the cost of producing grammatically disagreeing adjectives.

One might wonder how much these results reflect the “unmarked” nature of the masculine grammatical gender that, by default, is assigned in many languages (among which Italian) whenever specific information about the biological gender of the person is unknown or is thought to be irrelevant. However, the sentences clearly specified the grammatical gender of the referent since determiners were always marked for gender. So it seems unlikely that the masculine form of the adjectives was randomly provided in some cases but not in others.

In addition, it remains to be seen if indeed the masculine gender is generically intended (Corbett, 1991). Evidence showed that this is not always the case. McKay and Fulkerson (1979) showed that the use of the generic *he* led to a male-referent interpretation of antecedents such as, for instance, *student* or *musician*. More recently, Gygax et al. (2008) and Garnham et al. (2012) obtained similar results.

Finally, we conducted a qualitative analysis of the adjectives produced for a same metaphorical vehicle in the different experimental conditions. This analysis revealed that often participants produced the same adjectives, no matter the experimental conditions. Notwithstanding, a few interesting differences emerged. For instance, for the metaphorical vehicles *flagello* (*scourge*) and *incubo* (*nightmare*) the most frequent adjective was *severo* (*harsh*) when the subjects were *giudice* (*judge*) or *dirigente* (*manager*), both stereotypically male occupational nouns. But when the subjects were *vetrinista* (*window dresser*) or *igenista* (*hygienist*), both stereotypically female occupational nouns, the most chosen adjective in both cases was *incapace* (*unskilled*). This may reflect the different social status associated to these occupations, as revealed by the mean rating of social status provided in the Norming phase that were 6.35 for *judge*, 6.02 for *manager*, as opposed to 3.2 for *window dresser*, and 4.0 for *hygienist* (7 meant the highest social status and 1 the lowest).

This qualitative analysis did not reveal any effects of the concreteness/abstractness of the nouns used as metaphorical vehicles. However, this may depend on the fact that large part of them referred to abstract and emotional characteristics since we did not balance the abstract vs. concrete nature of the metaphorical meaning as in some other studies (e.g., Lecce et al., 2019). We also did not observe any overall significant differences depending on the male vs. female nature of the stereotyped role noun (for instance, more adjectives conveying the subject physical characteristics for female-oriented stereotypes than for male ones) but again this may reflect the experimental design of this study.

On more general grounds, the results of this exploratory study further confirm that indeed metaphorical meanings are pragmatically modulated by world knowledge, encyclopedic assumptions (Carston and Wearing, 2011) and, we showed, also by stereotypical beliefs when it comes to meaning availability. In addition, our results confirm previous claims about the evocative power of metaphor when referring to persons or social groups (Katz and Pexman, 1997; Hegstrom and McCarl-Nielsen, 2002; Maass et al., 2014; Ervas, 2017).

Admittedly, the results of this study provide more evidence about gender stereotypes than about the interplay between gender stereotypes and metaphors. This may reflect the lack of a condition containing literal descriptions of the same gender stereotyped and stereotype-neutral sentential subjects that, in principle, would have provided more fine-grained information about the specific contribution of metaphors. But would this literal condition indeed be possible and/or informative? By definition, metaphors are much more evocative than matched literal sentences; hence the interpretation of any possible differences would have been flawed by the communicative/cognitive non-equivalence of metaphorical and literal language (we return on this point below). Alternatively, one may have designed a study with the same experimental materials but using methods sensitive enough (e.g., Event-Related Brain Potentials) to capture differences in the moment-by-moment comprehension processes underlying the different experimental conditions (again we return on this point below). But this was beyond the aims of this exploratory study.

Clearly, this is an exploratory study and, as it is often the case for this type of study, it has important limitations. First, we did not directly compare (if indeed possible, as we said) the stereotypical knowledge encoded in literal and in metaphorical sentences. Hence we could not explicitly respond to the questions of whether metaphors are semantically equivalent and/or pragmatically more or less effective to matched literal sentences, and whether their power to shape attitudes is more effective than that of literal language. Further studies using different paradigms are necessary to directly respond to the question of whether a metaphoric framing of a stereotype indeed increases its impact. In addition, we cannot exclude that participants were reluctant to express their stereotypical attitudes explicitly listing more negatively valenced adjectives in one case or in the other. Studies also testing individual differences in stereotype sensitivity are necessary for a better understanding of the role of metaphors and gender stereotypes in describing people.

An intriguing question to which this study cannot respond concerns the relative time course of the activation of metaphorical meanings and social stereotypes. Recent evidence has shown that the stereotypical knowledge associated to stereotypical role nouns is immediately and automatically activated upon reading them (Mado Proverbio et al., 2018; Pesciarelli et al., 2019). This early activation may drive metaphor activation leading to the selection of the metaphor properties relevant to the topic, a process that may require time (for an overview on metaphorical meaning activation, see Bambini et al., 2016). Again, future studies are needed to assess the time course with which they are integrated during sentence comprehension.

Then, more females than male participants responded to this online study. Whether and how the biological gender of participants affects stereotypical attitudes is still a matter of discussion with mixed evidence concerning male-female differences (for an overview, see Canal et al., 2015; Fabre et al., 2016; Conrad and Von Scheve, 2017). In any case, a future study should balance the biological gender of participants to avoid possible gender biases.

Finally, assuming that listing the adjectives expressing the metaphor meaning equals to paraphrase its meaning, one might wonder whether metaphorical meanings are literally paraphrasable at all. The problem has a long story: Black (1979) claimed that a literal paraphrase “inevitably says too much – and with the wrong emphasis (.) – the loss in such cases is a loss in cognitive content”. Differently, Townsend (1988) defended the possibility of (successfully) paraphrasing familiar metaphors since one important function of paraphrase is to select from among multiple interpretations since paraphrasing a metaphor is an interpretive enterprise. No matter who is right, the adjectives produced by participants are interesting insofar as they reflect at least part of the interpretations assigned to metaphorical descriptions. If metaphors are ways for expressing the new with the old, the choice of which elements of our knowledge are used to name the “new” reveals the systems of beliefs and relevance implicitly adopted, for better or for worse.

## Data Availability Statement

The datasets generated for this study are available on request to the corresponding author.

## Ethics Statement

The studies involving human participants were reviewed and approved by the University of Modena and Reggio Emilia. The patients/participants provided their written informed consent to participate in this study.

## Author Contributions

CC conceived and planned the study, and was in charge of overall direction, and took the lead in writing the manuscript in consultation with EB. CC and EB contributed to the stimuli preparation and interpretation of the results. EB carried out the study and collected the data. EB analyzed the data.

## Conflict of Interest

The authors declare that the research was conducted in the absence of any commercial or financial relationships that could be construed as a potential conflict of interest.

## Footnotes

1 We also run two ANOVAs (respectively on the by-item mean percentage of grammatically correct adjectives and on grammatically disagreeing ones) with the factors Direction of stereotype (Male vs. Female) and Congruency (Congruent vs. Incongruent). Direction of stereotype was a between-item factor and Congruency a within-item factor. The ANOVA on correct adjectives revealed significant main effects of Congruency [*F*(1, 58) = 17.153, *p* = 0.0001, η^2^ = 0.232], and of the Direction of stereotype X Congruency interaction [*F*(1, 58) = 32.149, *p* = 0.0001, η^2^ = 0.357], but no main effect of Direction of stereotype [*F* < 1]. Similarly, the ANOVA on grammatically disagreeing adjectives showed significant main effects of Congruency [*F*(1, 58) = 15.754, *p* = 0.0001, η^2^ = 0.214], and of the Direction of stereotype X Congruency interaction [*F*(1, 58) = 38.94, *p* = 0.0001, η^2^ = 0.402], but no main effect of Direction of stereotype [*F* < 1].

## Appendix

### Experimental Word Pairs

**TABLE 8:** DELETE IT

**Male. Stereot.**	**Vehicle**	**Fem. Stereot.**	**Vehicle**	**Epicene**	**Vehicle**
Ambulante	ciclone	Costumista	ciclone	Persona	ciclone
Autista	libellula	Farmacista	libellula	Persona	libellula
Burocrate	cozza	Centralinista	cozza	Persona	cozza
Carabiniere	mostro	Babysitter	mostro	Persona	mostro
Concertista	veleno	Estetista	veleno	Persona	veleno
Consulente	ancora	Psicoterapeuta	ancora	Persona	ancora
Corriere	volpe	Fiorista	volpe	Persona	volpe
Deejay	bomba	Ginnasta	bomba	Persona	bomba
Dentista	scrigno	Linguista	scrigno	Persona	scrigno
Detective	macchina	Interprete	macchina	Persona	macchina
Dirigente	incubo	Igienista	incubo	Persona	incubo
Economista	diamante	Pediatra	diamante	Persona	diamante
Edicolante	vulcano	Contorsionista	vulcano	Persona	vulcano
Elettricista	fiore	Badante	fiore	Persona	fiore
Geometra	iceberg	Assistente	iceberg	Persona	iceberg
Giudice	flagello	Vetrinista	flagello	Persona	flagello
Giurista	camaleonte	Supplente	camaleonte	Persona	camaleonte
Immobiliarista	bocciolo	Telefonista	bocciolo	Persona	bocciolo
Ingegnere	pendolo	Alimentarista	pendolo	Persona	pendolo
Leader	trombone	Cartomante	trombone	Persona	trombone
Orefice	croce	Erborista	croce	Persona	croce
Paracadutista	roccia	Insegnante	roccia	Persona	roccia
Pilota	farfalla	Flautista	farfalla	Persona	farfalla
Pompiere	leone	Pallavolista	leone	Persona	leone
Preside	fossile	Callista	fossile	Persona	fossile
Sergente	avvoltoio	Promoter	avvoltoio	Persona	avvoltoio
Sommelier	scheletro	Dietista	scheletro	Persona	scheletro
Speaker	squalo	Logopedista	squalo	Persona	squalo
Urbanista	sonnifero	Spogliarellista	sonnifero	Persona	sonnifero
Vigilante	serpente	Colf	serpente	Persona	serpente
